# The activation of dormant ependymal cells following spinal cord injury

**DOI:** 10.1186/s13287-023-03395-4

**Published:** 2023-07-05

**Authors:** Francisco Javier Rodriguez-Jimenez, Pavla Jendelova, Slaven Erceg

**Affiliations:** 1 Stem Cell Therapies in Neurodegenerative Diseases Lab, Research Center “Principe Felipe”, C/Eduardo Primo Yúfera 3, 46012 Valencia, Spain; 2 National Stem Cell Bank - Valencia Node, Research Center “Principe Felipe”, C/Eduardo Primo Yúfera 3, 46012 Valencia, Spain; 3 Department of Neuroregeneration, Institute of Experimental Medicine, Czech Academy of Sciences, Prague, Czech Republic

**Keywords:** Spinal cord injury, Ependymal cells, Activation, Regeneration

## Abstract

Ependymal cells, a dormant population of ciliated progenitors found within the central canal of the spinal cord, undergo significant alterations after spinal cord injury (SCI). Understanding the molecular events that induce ependymal cell activation after SCI represents the first step toward controlling the response of the endogenous regenerative machinery in damaged tissues. This response involves the activation of specific signaling pathways in the spinal cord that promotes self-renewal, proliferation, and differentiation. We review our current understanding of the signaling pathways and molecular events that mediate the SCI-induced activation of ependymal cells by focusing on the roles of some cell adhesion molecules, cellular membrane receptors, ion channels (and their crosstalk), and transcription factors. An orchestrated response regulating the expression of receptors and ion channels fine-tunes and coordinates the activation of ependymal cells after SCI or cell transplantation. Understanding the major players in the activation of ependymal cells may help us to understand whether these cells represent a critical source of cells contributing to cellular replacement and tissue regeneration after SCI. A more complete understanding of the role and function of individual signaling pathways in endogenous spinal cord progenitors may foster the development of novel targeted therapies to induce the regeneration of the injured spinal cord.

## Background

Traumatic spinal cord injury (SCI) induces pathological processes that lead to severe and irreversible deficits, thus affecting a patient's physical, psychological, and social well-being and significantly impacting health-care systems worldwide. The functional restoration of damaged tissue after SCI represents a lofty yet fundamental goal in regenerative medicine.

Therapeutic strategies for SCI include the application of combinations of molecules/drugs, biomaterials, optogenetic approaches, three-dimensional bioprinting technology, and stem cell therapy to promote the regeneration of severed/damaged nerves [1]. The success of these strategies will be influenced by cell activity and communication within the niche after SCI, especially with resident stem cells whose activation, recruitment, and/or modulation could promote recovery. Stem cell transplantation represents a promising strategy for tissue regeneration after SCI, as these cells can survive post-administration and migrate to injured zones [2, 3]; however, critical challenges remain. For instance, preparing therapeutically relevant numbers of stem cells for autologous transplantation requires timespans that extend beyond the optimal treatment time window, while severe immune reaction or graft rejection also remains a significant concern related to allogenic stem cell transplantation [4, 5].

As previously mentioned, certain studies have validated the hypothesis that exogenous activation of endogenous progenitors represents a promising therapeutic strategy [6–8]; however, translating therapeutic strategies for SCI repair using ependymal cells from animal models to clinical studies should be approached cautiously. In humans of a certain age, injury can activate and stimulate dormant ependymal stem cells [9]; however, the central canal is mainly absent in the adult human spinal cord [2, 10]. Thus, the central canal in adulthood becomes substituted by a heterogeneous accumulation of astrocytes, ependymocytes, and perivascular pseudo-rosettes [10, 11]. Additional human studies will be crucial for characterizing the ependymal cell subset, their role after injury, and whether this NPC population possesses proliferative, migratory, and differentiation potential to contribute to spinal cord repair or plays a role in inhibiting/resolving glial scar formation with no regenerative potential.

### Ependymal cells from spinal cord in vivo

The existence of spinal cord-resident ependymal cells has incited interest in endogenous strategies as a therapeutic tool [12, 13]. Radial glial cells, specialized cells in the developing nervous system, serve as primary progenitor cells in the central nervous system (CNS) and differentiate into ependymal cells (the possible endogenous neural stem cells [NSCs] of the adult spinal cord [13]) and astrocytes during early postnatal periods in mammals [14]. Interestingly, a subpopulation of radial glial cells possesses deuterostomes, which participate in the early development of cilia, thereby reinforcing radial glia as ependymal cell progenitors [15]. The ependymal cells that line the spinal cord's central canal [16] possess characteristic cilia that move cerebrospinal fluid. A subependymal layer containing small numbers of ciliated astrocytes, oligodendrocyte progenitors, and neurons borders the ependymal cells. Ependymal cells also form the neurogenic niche of the adult brain's subventricular zone (SVZ) in combination with dividing glial fibrillary acidic protein (GFAP)-expressing cells (B1 astrocytes), which take on a pinwheel-like cytoarchitecture [17]. Monociliated B1 astrocytes form the core of pinwheels with NSCs-like characteristics, which are surrounded by cells with complex basal bodies with long cilia (often biciliated) comparable to ependymal cells [17]. The ependymal layer in spinal cord tissue possesses a less elaborate form than the adult SVZ [18, 19], taking on a "pearl necklace"-like appearance with bi- or multi-ciliated cells [18]. The most common ependymal cell type line the central canal of the spinal cord and possess two long motile cilia; however, studies have also described bi-nucleated cells with four cilia and cells with one or three cilia associated with large basal bodies [18]. Additional cell subpopulations within the spinal cord central canal, such as GFAP-expressing astrocytes with a single cilium, display similarities to the B1 astrocytes of the SVZ [18].

**Fig. 1:**
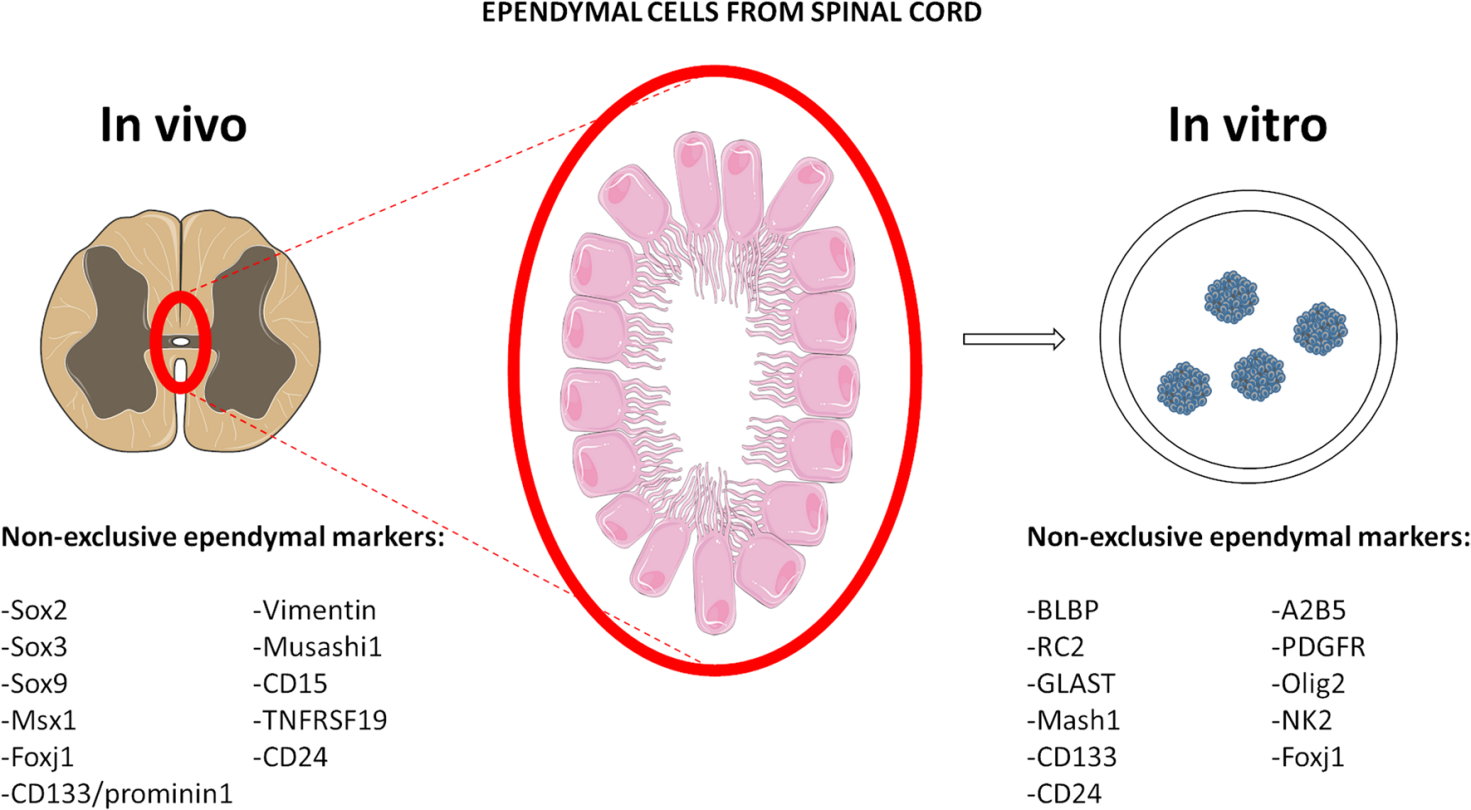
Ependymal cells from spinal cord. Ependymal cells in central canal are marked in red in a transversal section of spinal cord and amplified for better view. Neurospheres from homogenized spinal cord tissue can grow in defined conditions. Commonly accepted but non-exclusive markers of ependymal cells in vivo and in vitro are listed accordingly

Spinal cord-resident ependymal cells are morphologically and molecularly heterogeneous and may reside in different cell subpopulations [10, 20, 21]. Widely accepted, but not exclusive ependymal cell markers include SRY-box transcription factor 3 (Sox3), Sox9, CD15, CD133/prominin1, vimentin, Musashi1, CD24 [21], and Forkhead Box J1 (FoxJ1) [3] (Fig. 1). While only dorsally positioned ependymal cells express nestin/GFAP, all ependymal cells express the Sox2 transcription factor [12, 20, 22]. Ki67 expression (in addition to Nestin and Sox2) specifically characterizes the ependymal cells observed in injured tissue [23]. Given the heterogeneity within the NSCs pool in the spinal cord [13], several recent studies sought to identify ependymal cell subpopulations with NSCs-like properties. Some studies identified cells expressing Msh homeobox 1 (Msx1) in a quiescent state [24] and Troy, TNF receptor superfamily member 19 (TNFRSF19) in an activated state [25]. Single-cell RNA sequencing (scRNA-seq) experiments have revealed that activated ependymal cells gain stem cell features after injury before differentiating to astrocyte- or oligodendrocyte-lineage cells [25]. Additional studies demonstrated that DNGR-1 expression marks a population of ventricular progenitors committed to an ependymal cell subset endowed with damage-responsive NSCs potential in adulthood. DNGR-1-traced ependymal cells possess latent regenerative potential and mobilize in response to local injury [26].

The stem cell potential of ependymal cells in the adult spinal cord remains controversial. Neural stem cell progeny exerts a neurotrophic effect required for survival of neurons adjacent to the lesion and is required for maintaining the integrity of the injured spinal cord [3]. Advances in lineage tracing and single-cell sequencing technologies have supported reports of the low contribution of ependymal cells to activated cells after SCI [27, 28]. Therefore, exploring whether ependymal cell activation represents a feasible approach to spinal cord repair after injury must also decipher the molecular mechanisms involved in activation, which extends into the first few weeks post-SCI [29]. This knowledge may contribute to the development of novel therapeutic strategies in regeneration after SCI and the enhanced function of the endogenous regenerative machinery, thereby constituting an exciting alternative or complementary approach to cell transplantation strategies. Exogenous stem cells transplanted into the spinal cord of athymic rats or rat models of motor neuron disease induce endogenous stem cell activity and initiate intrinsic repair mechanisms [30]; however, whether transplanted cells favor paracrine activity and neurotrophic support, with possible positive effects on the endogenous ependymal cell population after SCI, remain unexplored. Recent research has shown that human fetal brain-derived NSCs and NPCs, embryonic stem cell-derived NPCs, and spinal cord-derived NSCs (ependymal cells) possess similar gene expression profiles; however, some differences have been observed. For instance, ependymal cells over-expressed a greater proportion of genes related to nerve function [7], while ependymal cells displayed enhanced survival and a greater propensity to differentiate into neurons after transplantation in the SCI in rats [7]. In vivo comparisons have also underlined the optimal therapeutic effects of ependymal cells, which included electrophysiological and hindlimb functional recovery [7]. On the other hand, recent studies have raised doubt regarding the status of the spinal cord ependymal region as a neurogenic niche and its involvement in cell replacement after lesions in adult humans [11, 18].

The microenvironmental conditions of the spinal cord may influence the previously noted poor neurogenic capacity of ependymal cells [31]. In fact, cloned and expanded adult spinal cord ependymal cells can generate neurons and glia after transplantation into the adult rat dentate gyrus when exposed to the appropriate microenvironment [32]. Cell transplantation in the spinal cord injury site could induce enhanced microenvironmental conditions that support spinal cord regeneration [33]; however, the inhibitory microenvironment that develops after SCI often causes transplanted NSCs and endogenous ependymal cells to differentiate into glial cells rather than neurons [34]. To avoid these adverse effects, certain studies have attempted to improve microenvironmental conditions after SCI to promote the differentiation of exogenous and endogenous NSCs into neurons [35–37]. Niche activation via pharmacological agents could suffice to create a protective environment for newborn neurons [8, 38] or NSCs transplanted into the spinal cord, which could induce functional improvement in mice after injury [8, 39]. Therefore, enhancing the neurogenic potential of an ependymal cell or modifying the microenvironment represents an attractive strategy in SCI-focused regenerative medicine.

Epigenetic events can influence the in vivo expression of certain genes in ependymal cells in spinal cord tissue after SCI. Histone acetylation and DNA methylation are epigenetic modifications responsible of regulating patterns of gene expression and are crucial under normal physiological conditions, during development and pathological conditions. An analysis of transcriptional responses of neuronal and ependymal populations after SCI showed that are shaped by histone deacetylase 3 (HDAC3) activity [40]. Based on the expression of ependymal markers Sox2, Foxj1, and regulatory subunit Of Type II PKA R-subunit domain containing 1 (Riiad1), the authors showed 1838 differentially expressed genes between ependymal cells and neurons. HDAC3 affected transcription of 448 genes in ependymal cells, with the majority (78%) repressed by HDAC3, obeying the general principle of gene repression caused by histone deacetylation. Authors showed that SCI caused a marked ependymal expansion with a substantial contraction of neuronal populations. The affected genes were connected to ECM organization, voltage-gated ion channels, as well as Wnt, Hedgehog, and platelet-derived growth factor B signaling. These results indicate a reactive gene profile of ependymal cells after injury [40]. Thus, defining the epigenetic mechanisms that regulate progenitor cells after SCI would help to design strategies to promote the contribution of ependymal cells in wound healing and tissue preservation after injury.

### Ependymal cells from spinal cord in vitro

**Fig. 2:**
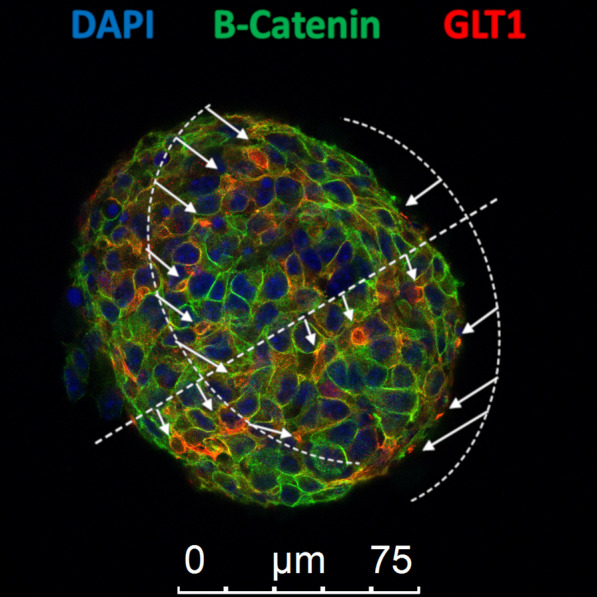
In vitro organization of ependymal stem cells grown as neurospheres. GLT-1 antibody was used as marker of V-SVZ astrocytes, type B1 positioned at the core of the pinwheel structure (red), and a β-catenin antibody to delineate cell borders showing big cells in its periphery, presumably ependymal cells (green). DAPI was used to mark nuclei (blue). Arrows indicate pinwheel´s cores and dashed lines their alignments. Scale bars: 75 µm (unpublished data)

While we still lack a complete understanding of the organization of ependymal cells grown as neurospheres in vitro [41], our laboratory recently reported the formation of pinwheel structures in spinal cord- and SVZ tissue-derived neurospheres cultured in vitro [42]. Neurospheres are widely used in vitro three-dimensional culture system composed of free-floating clusters of proliferating neural stem cells. We have shown that this organotypic-like culture resembles the neurogenic niche organization of the adult SVZ. The pinwheel’s core contains the apical endings of B1 cells and in its periphery is consisted of the ependymal cells [17]. We showed the presence of pinwheels in neurospheres obtained from spinal cord [42]. We observed the alignment of these cores with apparent equidistant position, in a well-organized manner that may contribute to form round neurospheres (Fig. 2) (unpublished data). Neurospheres may offer an opportunity to study neurogenic mechanisms under normal or pathological conditions, thereby opening new perspectives for therapeutic interventions. Heterogeneous in vitro neurosphere cultures of ependymal cells obtained from the SVZ, olfactory bulb, and spinal cord [43, 44] revealed the expression of markers for radial glia (e.g., brain lipid-binding protein [BLBP], radial glial cell marker 2 [RC2], and glutamate/aspartate transporter [GLAST]). In parallel, it has been observed the expression of oligodendrocytes/neurons markers (e.g., achaete-scute complex-like 1 [Mash1], oligodendrocyte transcription factor 2 [Olig2], NK2 homeobox 2 [Nkx2.2], neural/glial antigen 2 [NG2], neuron cell surface ganglioside epitope [A2B5], and platelet-derived growth factor receptor [PDGFR]) [45], (Fig. 1). Notably, primary neurospheres derived from ependymal cells and grown in vitro exhibit limited self-renewal capacity [20, 46, 47]. However, a significant increase in proliferation activity was observed in vitro of neurospheres derived from ependymal cells isolated from rats 1 week after SCI as compared with cultures obtained from non-injured control rats. This means that in vitro neurosphere forming potential of ependymal cells increases after SCI [2, 48]. These cells we named induced ependymal cells. Interestingly, telomerase activity was augmented in these cells as well as the expression levels of both Sox2 and Oct4, factors which are critical for pluripotency, and self-renewal when compared to ependymal cells derived from uninjured animals. These activated (or induced) ependymal cells have capacity to differentiate into astrocytes and oligodendrocytes under defined in vitro conditions, although they lack robust neurogenic potential [12, 13, 32, 48–50]. It is crucial to determine the variety of molecular signals involved in activation of ependymal cells after SCI suggests that these mechanisms might be exploited to repair spinal cord. On the other hand, certain epigenetic mechanisms regulate the expression of proteins with a relevant presence in ependymal cells when grown as neurospheres in vitro.

Epigenetic regulation was observed in the expression of the ependymal marker *FoxJ1* that was silenced by methylation of a CpG island. The forced DNA demethylation by treatment with 5-azacytidine (5-aza-dc) rescues *FoxJ1* mRNA expression in neurospheres obtained from spinal cord [42]. This hint suggests that epigenetic mechanisms can regulate expression of FoxJ1 a crucial protein with a role in an ependymal niche organization [51].

### Ependymal cells from injured spinal cord transplanted in vivo

We have already shown that isolation of induced ependymal cells from SCI rats and subsequent transplantation in contusion models showed long-distance migration from the transplant site to the lesion zone participating in the improvement of functional locomotor recovery [2, 52]. These studies suggest that the ependymal cells undergo phenotypic and genotypic changes after SCI such as increase in self-renewal properties, better response to differentiation signals, and improved regenerative capacity. The study of Ohori et al. [53] showed the possibility to manipulate the neuronal and glial differentiation of endogenous NPCs in vivo. They give the evidence that overexpression of the proneural transcription factors Neurogenin2 and Mash1 together with treatment with growth factors stimulate neurogenesis and oligodendrogenesis, when injected into the injured spinal cord. Mobilizing endogenous NPCs and forced differentiation by growth factor treatment and genetic manipulations may lead to the development of novel cell replacement therapy for SCI.

## Expression of SCI non-exclusive markers in ependymal cells after SCI

The confined nature of NSCs activity to spinal cord-resident ependymal cells has provided interest in this cell population as a therapeutic tool [12, 13, 46]. As mentioned above, ependymal cells in the intact spinal cord display limited self-renewal capacity [47]; thus, ependymal cells represent dormant cells in the intact spinal cord that becomes activated by injury [23]. While a general and rapid loss of neural cell types, including stem/progenitor cells, occurs 1 day after SCI [54], surviving endogenous ependymal cells become activated in response to distinct types of injury by triggering injury-specific molecular events [29]. Studies have reported that resident ependymal cells positively contribute to spinal cord regeneration by neurotrophic support, impairing cyst formation, or restricting the extent of secondary injury processes [3, 46]. Ependymal cells may also contribute to glial scar formation after SCI [55] but with minimal/local contribution and depending on direct damage in the ependymal layer [56]. Regardless of the injury type, an early increase in proliferation represents a typical cellular response in ependymal cells after injury [2, 54, 57, 58] followed by migration toward the injury site [13, 16, 58].

While a range of factors contributes to molecular changes in ependymal cells after SCI, we briefly discuss the most critical transcription factors, cell adhesion/extracellular matrix molecules, receptors, and ion channels that may represent future targets for the therapeutic modulation of ependymal cells in the following chapters (Table 1).

**Table 1:** Changes of mRNA and protein expression in ependymal cells from injured spinal cord and grown as neurospheres in vitro

Gene symbol	Species	Expression	Reference(s)
P2Y1	Rat	↓ mRNA and prot	[79]
P2Y4	Rat	↑ mRNA and prot	[79]
Cx37	Rat	↓ mRNA and prot	[98]
Cx40	Rat	↓ mRNA and prot	[98]
Cx43	Rat	↓ mRNA and prot	[98]
Cx50	Rat	↓ mRNA and prot	[92, 98]
NCAM	Rat	↑ mRNA	[2]

Gene symbol	Species	Expression/time	Reference (s)
Changes of protein expression ependymal cells in injured spinal cord (central canal) in vivo			
BAF45D	Rat	↓ prot/10–14 dpi	[59]
ATF3	Rat	↑ nuclear prot/6–24 h	[60]
Sox9	Mouse	↑ prot/2 w–10 mo	[13]
Β-Integrin	Mouse	↑ prot/2 dpi	[71]
GPR17	Mouse	↑ prot/72 h–1 w	[77]
Cx26	Mouse	↑ prot/5 dpi	[99]
Cx50	Mouse	↓ prot/1 w	[98]
OSMR	Mouse	↑ mRNA and prot/72 h	[61]
FoxJ1	Mouse	↓ prot/72 h	[61]
CXCR4	Mouse	↓ prot/5 w	[74]
NCAM	Rat	↑ prot/1–3 dpi	[72]

Gene symbol	Species	Expression/time	Reference (s)
Changes of protein expression in injured spinal cord in vivo within the region of hosted ependymal cells after transplantation			
P2X4	Rat	↓ prot/2 mo	[79]
P2X7	Rat	↓ prot/2 mo	[79]
Cx50	Rat	↓ prot/2 mo	[92]

Messenger ribonucleic acid, (mRNA); protein, (prot). Days post-injury, dpi; hours, h; week, w; and month, mo

### Transcription factors

After SCI, ependymal cells display well-orchestrated alterations to the expression of transcription factors involved in significant events such as cell viability, division, differentiation, migration, and cilia formation [59–61]. Within 3 days after injury, ependymal cell progeny leaves the central canal region and migrates toward the injury site [13, 16, 58]. The process of migration toward the injury may associate with the loss of ependymal phenotype, as judged by the reduced expression of typical transcription factors associated with ependymal cells such as Sox2, Sox3, and FoxJ1 [13]. Ependymal cells microdissected from the central canal region 72 h after SCI in mice display an increase in the expression of cilia-associated transcription factor regulatory factor X4 (Rfx4), but a decrease in other factors such as regulatory factor X1 (Rfx1), tumor protein P53 (Trp53), and FoxJ1 [61]. In adult rats, the downregulated expression of the BAF45D transcription factor (BRG1-associated factor 45D) in ependymal cells following SCI [59] correlates to the inhibition of neuronal differentiation, which acts indirectly by reducing the pool of neural progenitor cells (NPCs). This study partially supports the role of BAF45D in SCI-related neuropathology [59]. The basic leucine zipper activating transcription factor 3 (ATF3), a critical transcription factor in axon regeneration, becomes rapidly upregulated in injured neurons after peripheral injury [62]. ATF3 overexpression may contribute to neurite outgrowth by orchestrating alterations to gene expression in injured neurons [63], thereby contributing to spinal cord regeneration [64]. ATF3 expression in ependymal cells from injured rats overlaps with nestin, vimentin, and Sox2 expression [60] while migrating ependymal cells from injured rats express both Sox9 and ATF3 [13, 60, 65]. Immunohistological analysis of dissected spinal cords demonstrated the importance of ATF3 localization; in this study, ATF3 translocated from the cytoplasm of ependymal cells to the nucleus after activation and mobilization [60]. Thus, ATF3 could represent a reliable marker of activated NPCs in the rat spinal cord [60].

Olig2 belongs to the b-HLH transcription factor family and plays relevant roles during CNS development and regulates remyelination in models of demyelination CNS disorders. Although Olig2 expression in ependymal cells increases following SCI, scar tissue formation at later time points fails to induce Olig2 expression, which is restricted to uninjured tissue bordering the scar [13]. Although the role of Olig2 in SCI and any possible therapeutic impact remain elusive, evidence suggests that ependymal cells from injured animals may contribute to the formation of myelinating oligodendrocytes to improve functional recovery [60, 65]. Latent lineage potential resident in NSCs enabled SCI repair; the authors reported that expression of OLIG2 in ependymal cells leads to the activation of the latent oligodendrocyte-lineage program, which could support the recovery of axon conduction after injury [66].

### Cell adhesion and extracellular matrix molecules

Ependyma cells express cell adhesion molecules (e.g., E-cadherin, β1-integrin, and neural cell adhesion molecule [NCAM]) and extracellular matrix proteins (e.g., fibronectin, laminins, thrombospondin 2 [THBS2], and chondroitin sulfate proteoglycans [CSPGs]) from different subfamilies; however, their functions after SCI remain incompletely understood [67].

Cell adhesion molecules help maintain ependymal cell architecture, shape, survival, proliferation, and differentiation in the stem cell niche and support migration [67, 68]. While studies of E-cadherin have failed to demonstrate any differences in expression between control and SCI-affected dogs at the lesion epicenter or proximal sites [69], differences exist in the subcellular distribution of E-cadherin after SCI. While E-cadherin exclusively localizes to the apical section of ependymal cells in uninjured dogs, SCI induces the re-localization of E-cadherin to the cytosol and circumferential membrane [69]. Taking into account that overexpression of E-cadherin facilitates motor function recovery following SCI by reducing the release of inflammatory cytokines in case of transplanted NSCs [70], future investigation is needed to determine whether E-cadherin could be efficient target in ependymal cells for regenerative purposes. β1-integrin also becomes robustly upregulated in ependymal cells following SCI, probably to induce ependymal cell migration to the injury site [71]. β1-integrin expression by ependymal cells may play a critical role in astrocytic differentiation of ependymal cells in vivo following SCI by helping to maintain the stem/progenitor cell state. A study by North et al. demonstrated that ablation of β1-integrin expression in ependymal cells decreased the levels of stem cell markers in progeny but increased GFAP expression and astrocytic differentiation [71]. Thus, the β1-integrin signaling system constitutes a potential therapeutic target to modify astrogliosis and limit the detrimental effects of glial scar formation after SCI [71]. Moreno-Manzano et al. [2] reported increased *NCAM* expression in in vitro cultures of induced ependymal cells from SCI rats compared to ependymal cells from uninjured rats; however, how NCAM influences ependymal cell behavior after SCI remains unknown. In complete transaction spinal cord animal model, the expression level of NCAM is markedly elevated at 1 day and 3 days post-injury and strongly expressed in in motor neurons (3 days post-transection) and in dorsal sensory and corticospinal fiber tracts (8 days post-transection) [72]. Bearing in mind, the role of NCAM in mediating cell migration, survival, neurite growth and synaptic plasticity [73], and its possible correlation with functional recovery of the spinal cord [72] suggesting a role for this protein in pathological development after SCI [72]. By generating a molecular resource through RNA profiling of ependymal cells before and after injury, Chevreau et al. [61] observed the upregulated expression of the adhesive glycoprotein *THBS2*, besides the other signaling pathways. THBS2 has a role in mediating cell-to-cell and cell-to-matrix interactions and might contribute to the termination of post-trauma angiogenesis but there is little information concerning whether this molecular change could have relation to ependymal cell activity after injury.

### Receptors and ion channels

The expression of specific receptors in the neurogenic niches of adult rodents and humans, including the central canal of the spinal cord, regulates stem cell responses after SCI.

Analysis of ependymal cells obtained by tissue laser microdissection after SCI also established an increase in oncostatin M (OSM) receptor (OSMR) [61]; furthermore, the same study reported OSM (inflammatory cytokine)-induced robust OSMR expression in spinal cord-derived neurospheres. This study shows that the OSM/OSMR pathway may regulate ependymal cell proliferation and differentiation, particularly the astrocytic fate of ependymal cells after SCI. Studies have indicated that stromal cell-derived factor 1 (SDF-1) plays a vital role in the chemotaxis of stem cells through an interaction with chemokine receptor C-X-C motif chemokine receptor 4 (CXCR4). Tysseling et al. observed CXCR4 in the ependymal cells surrounding the central canal; however, the CXCR4 expression pattern in the spinal cord altered 5 weeks after SCI with reduced CXCR4 in the ependymal layer [74]. The reduced expression of this receptor by ependymal cells after injury suggests that CXCR4 does not control the migration of ependymal cell progeny toward the injury site [13]; instead, the SDF-1/CXCR4 axis may promote recovery after SCI by mediating the migration and attraction of bone marrow-derived mesenchymal stem cells [75].

#### Purinergic receptors

Response to CNS injury also involves purinergic (P2) receptors [76]; however, the detailed contribution of P2 receptors and G protein-coupled receptors (P2Y) and ligand-gated ion channels (P2X) in ependymal cells under in vitro and in vivo conditions remains elusive. The metabotropic P2Y receptors respond to signaling molecules or agonists such as adenine and uridine nucleotides (ATP, ADP, UTP, and UDP) and nucleotide sugars (UDP-glucose). G protein-coupled receptor 17 (GPR17), a P2Y-like receptor responding to both uracil nucleotides (e.g., UDP-glucose) and cysteinyl-leukotrienes, is normally expressed by a subset of neurons, oligodendrocytes, and ependymal cells lining the central canal but not astrocytes [77]. A study by Boccazzi et al. determined that P2Y-like GPR17 receptor could modulate the multipotency of oligodendrocyte precursor cells in vitro [78]. GPR17 may function as a damage “sensor,” becoming activated by nucleotides and cysteinyl leukotrienes released in the lesioned area; however, GPR17 could also participate in post-injury responses. Ceruti et al. proposed an interesting dual and spatiotemporal-dependent role for GPR17 after SCI [77]. They discovered that GPR17-mediated neuronal and oligodendrocyte death within the lesion early after injury; however, the injection of a specific GPR17 antisense oligonucleotide into the spinal cord impaired cell death and significantly ameliorated SCI-induced tissue damage and motor deficits. At later phases after injury, GPR17 may participate in beneficial remodeling and repair activated by danger signals through microglia/macrophages recruitment from distal parenchymal areas and move toward the lesioned zone. The induction of the astrocytes cell marker GFAP in GPR17-expressing ependymal cells suggested the initiation of repair mechanisms [77]. Overall, these findings provide evidence for the designation of GPR17 as a target for therapeutic manipulation to promote remyelination and functional repair in SCI.

Ependymal cells grown as neurospheres in vitro respond to changes in ATP, ADP, and other nucleotides by activating the ionotropic P2X4 and P2X7 and metabotropic P2Y1 and P2Y4 purinergic receptors [79]. Activation of ependymal cells by SCI downregulates the expression of the P2Y1 receptor and upregulates the expression of the P2Y4 receptor [79]. The increased expression of the P2Y4 receptor in ependymal cells after injury could facilitate the expansion of mitotic neural precursors in vitro*,* while a decrease in P2Y1 could favor neuronal/glial differentiation [52]. Ependymal cells from the intact rat spinal cord do not express functional P2Y2 receptors, as demonstrated by the poor calcium (Ca^2+^) response to the agonist Ap4A [52]; however, P2Y2 receptor expression becomes altered in rats after SCI [80]. This study analyzed the spatiotemporal expression of P2Y2 receptors in the spinal cord after SCI and demonstrated a significant increase in P2Y2 mRNA between 2- and 28-day post-injury. Ionotropic P2X receptors, membrane ion channels permeable to sodium (Na^+^), potassium (K^+^), and Ca^2+^ open within milliseconds of ATP binding [81–83]. The effects of purinergic agonists on ependymal cells in the neonatal rat spinal cord suggest that P2X7 ion channel receptors and downstream cellular events (e.g., Ca^2+^ waves) represent possible targets to manipulate the response of the ependymal cell niche to ATP released after SCI [84, 85]. Highly selective pharmacological inhibition of P2X7R in rats by the administration of adenosine 5'-triphosphate-2',3'-dialdehyde (OxATP) [86] or Brilliant Blue G (an analog of a commonly used food additive with low toxicity) [87, 88] reduces tissue damage and improves motor performance after SCI [89]. The previous reports suggested an early and persistent increase in P2X4 and P2X7 [79] receptor expression around the injury site after severe spinal cord contusion in rats; however, the transplantation of ependymal cells from injured animals in the rat SCI model reversed the increase in P2X4 and P2X7 expression [79]. Related reports have noted the need for further preclinical investigations before evaluating the inhibition of the P2X7 receptor as a treatment for contusive SCI in clinical trials [90].

#### Connexin ion channels

Connexins (Cx) comprise a large family of transmembrane proteins that function in gap junction intercellular communication. Besides docking with connexins in neighboring cells, these ion channels form "hemichannels" or "connexons" that exist independently within an individual cell [91]. Recently, the role of these ion channels in crucial stem cell-related processes, including self-renewal and differentiation, has become increasingly prominent [92, 93]. Importantly, connexins also play relevant roles in spinal cord physiology and functional recovery after SCI [94, 95].

Cx43 is the most widely studied connexin, contributing to the secondary expansion of traumatic SCI and playing a vital role in neuropathic pain [96]. Administration of a Cx43 mimetic peptide after SCI in rats prompted a reduction in tissue damage and improved functional recovery, which might relate to the inhibited pathological opening of Cx43 hemichannels [97]. Ependymal cells obtained from injured rats and cultured in vitro exhibited the downregulated expression of Cx37, Cx40, Cx43, and Cx50 compared to ependymal cells from uninjured rats [98]. Interestingly, the plasma membrane represents the most frequent cell location for connexins; however, the location of Cx50 within the nucleus of ependymal cells [98] and astrocytes [92] suggests a potential role for this ion channel beyond cell-to-cell communication. A recent study reported that ependymal cell coupling increased after injury, paralleled by the upregulated expression of Cx26 [99]; however, Cx26 blockade reduced the injury-induced proliferation of ependymal cells. The authors suggest the altered expression of connexins as an early feature of ependymal cells after SCI, which may represent a target to improve the contribution of the central canal stem cell niche to repair [99].

Purinergic receptors and connexins are closely related families of cell membrane proteins that interact to coordinate molecular events in specific CNS cells to sense injury and activate suitable responses. Under pathological conditions such as SCI, glial activation depends on the communication between neurons and astrocytes mediated by connexin, pannexin, and purinergic receptors [100]. Suadicani et al. discovered that the acute downregulation of Cx43 in mouse spinal cord astrocytes caused the decreased expression of the P2Y1 receptor and increased expression of the P2Y4 receptor [101, 102]. Other studies have reinforced the idea of a relationship between connexins and P2 receptors in NSCs. For instance, the reduced expression of P2Y1 receptors in Cx43-null mice alters Ca^2+^ signaling and NPC migration [82]. Transplantation of ependymal cells from injured rats in host animals reversed the increased expression of *P2X4* and *P2X7* receptors [79] and Cx50 [92] in the grafted region around the SCI. The absence of Cx50 expression in grafted ependymal cells from injured rats suggested a minor regenerative role or detrimental contribution of this ion channel to stem cell engraftment [92]; however, whether connexins and purinergic receptors function together to contribute to a more permissive environment for axon growth and cell survival after transplantation of NSCs remains to be elucidated.

## Conclusions

An orchestrated modulation of gene expression profiles (affecting transcription factors, cell adhesion molecules, receptors, and ion channels) occurs during the transition of ependymal cells from uninjured animals into activated ependymal cells after SCI. Understanding the regulation of expression of these genes in ependymal cells in vitro and in vivo may provide new insight into parallel in vivo processes occurring after injury. Many studies in animal models have shown that injury-activated ependymal cells could contribute to the regenerative process [2]; however, the view of the spinal cord ependymal region as a neurogenic niche in adult humans remains under doubt due to the results from studies suggesting the lack of involvement of these cells in cell replacement processes after injury [11, 27, 103]. The various studies performed in different species and models and the requirement of detailed tracking studies to determine the origin and fate of cells before and after SCI may partially explain this controversy. This review summarized the main factors involved, including transcription factors and receptors, whose expression becomes significantly modulated in ependymal cells after SCI. Of note, the modulation of cell responses, such as ependymal cells after SCI, could avoid the exacerbated responses that contribute to secondary injury (e.g., inflammation and reactive oxidative damage) and undesired effects (such as pain). Pharmacological intervention to control the activation of the resident stem cells in the spinal cord represents a significant challenge to developing safe and efficient SCI repair/regeneration strategies.

## Data Availability

Not applicable.

## Abbreviations

SCI: Spinal cord injury
CNS: Central nervous system
NSCs: Neural stem cells
SVZ: Subventricular zone
scRNA-seq: Single-cell RNA sequencing
ECM: Extracellular matrix
CpG Island: Cytosine and guanine in a repeated sequence
DNA: Deoxyribonucleic acid
RNA: Ribonucleic acid
GFAP: Glial fibrillary acidic protein
GLT1: Glutamate transporter-1
Sox3: SRY-box transcription factor 3
Sox9: SRY-box transcription factor 9
Foxj1: Forkhead box J1
Msx1: Msh homeobox 1
TNFRSF19: TNF receptor superfamily member 19
Ki-67: Marker of proliferation Ki-67
HDAC3: Histone deacetylase 3
WNT: Wingless-related integration site
Riiad1: Regulatory subunit of type II PKA R-subunit domain containing 1
BLBP: Brain lipid-binding protein
RC2: Radial glial cell marker 2
GLAST: Glutamate/aspartate transporter
Mash1: Achaete-scute family b-HLH transcription factor 1
b-HLH: Basic helix-loop-helix transcription factor
Olig2: Oligodendrocyte transcription factor 2
Nkx2.2: NK2 homeobox 2
NG2: Neural/glial antigen 2
A2B5: Neuron cell surface ganglioside epitope
PDGFR: Platelet-derived growth factor receptor
Oct4: POU class 5 homeobox 1
5-Aza-dc: 5-Azacytidine
Rfx4: Cilia-associated transcription factor regulatory factor X4
Rfx1: Cilia-associated transcription factor regulatory factor X1
Trp53: Tumor protein P53
BAF45D: Transcription factor (BRG1-associated factor 45D)
DPF2: Double PHD fingers 2
ATF3: Activating transcription factor 3
NCAM: Neural cell adhesion molecule
THBS2: Thrombospondin 2
CSPGs: Chondroitin sulfate proteoglycan
OSM: Oncostatin M
SDF-1: Stromal cell-derived factor 1
CXCR4: Chemokine receptor C-X-C motif chemokine receptor 4
P2 receptors (P2Y, P2X): Purinergic receptors
ATP: Adenosine triphosphate
ADP: Adenosine diphosphate
UTP: Uridine triphosphate
UDP: Uridine diphosphate
GPR17: G protein-coupled receptor 17
Ca2+: Calcium
Na+: Sodium
K+: Potassium
OxATP: Adenosine 5'-triphosphate-2',3'-dialdehyde
Cx: Connexins
DAPI: 4',6-Diamidino-2-fenilindol
